# Pt-enhanced WO_3_ nanoparticles for efficient hydrogen production: synthesis and electrochemical evaluation

**DOI:** 10.55730/1300-0527.3734

**Published:** 2025-03-18

**Authors:** Merve AKBAYRAK

**Affiliations:** Department of Biotechnology, Faculty of Science, Necmettin Erbakan University, Konya, Turkiye

**Keywords:** Hydrogen evolution reaction, electrocatalyst, platinum, tungsten oxide, electrochemical water splitting, metal oxides

## Abstract

In this study, Pt/WO_3_ nanoparticles were synthesized using a simple room-temperature impregnation-reduction method and characterized by advanced techniques including ICP, TEM-EDX, FE-SEM-EDX, and XRD. The ICP-OES analysis confirmed a 1.0 wt. % Pt loading on the WO_3_ support. TEM and FE-SEM analyses revealed that the Pt nanoparticles were well dispersed with an average size of approximately 3.7 nm. The XRD patterns showed characteristic WO_3_ peaks without any detectable Pt diffraction peaks, indicating the high dispersion of Pt. Electrochemical evaluations demonstrated that the Pt/WO_3_ catalyst exhibited outstanding hydrogen evolution reaction (HER) performance, with −27.8 mV vs. RHE onset potential and −37.4 mV overpotential at 10 mA.cm^−2^, outperforming bare WO_3_. The Tafel slope (b) of 68.6 mV·dec^−1^ indicates efficient reaction kinetics following the Volmer-Heyrovsky pathway. The impedance analysis confirmed efficient charge transfer, with a b value of 69.7 mV.dec^−1^. The ECSA was calculated as 8.575 cm^2^, highlighting the high surface activity of the catalyst. Stability tests showed minor degradation but retained significant catalytic activity. This work emphasizes the potential of Pt/WO_3_ as an environmentally friendly, cost-efficient catalyst with promising applications in HER, providing a scalable and effective approach to hydrogen production.

## 1. Introduction

Water splitting is important for addressing energy issues as it facilitates the generation of clean and sustainable hydrogen fuel [1]. Hydrogen is an important energy carrier and which has applications in diverse fields, including power generation, industry, and transportation [2]. Utilizing renewable energy sources for water splitting enables hydrogen production without emitting greenhouse gases, thereby reducing reliance on fossil fuels and supporting a transition to sustainable energy. Additionally, hydrogen can be transported and stored, making it a valuable energy storage solution. This further supports the integration of intermittent renewable energy sources into the energy system [3]. Although the H_2_ evolution reaction (HER) is central to hydrogen production, its widespread implementation encounters challenges such as the need for catalysts made of costly and scarce materials, slow reaction kinetics, and the requirement for large energy inputs, necessitating ongoing research to improve efficiency, lower costs, and advance the scalability of HER-based hydrogen production technologies [4]. Platinum (Pt) is a commonly used catalyst for HER because of its outstanding catalytic performance and stability [5].

The volcano plot for the HER shows the relationship between the catalytic activity of different catalysts and their binding energy for hydrogen [6]. It suggests that the optimal catalyst for the HER should have an intermediate binding energy. Pt is considered the benchmark catalyst and exhibit excellent activity for the HER [7]. It is positioned at the summit of the volcano plot, signifying its excellent activity for the HER [8]. Pt has an optimum adsorption strength for hydrogen, and it facilitates both hydrogen atoms’ adsorption and desorption during the reaction [9]. However, Pt suffers from low abundance and high cost which limits widespread use of Pt in large-scale applications [10]. Researchers are actively working on finding alternative catalysts that exhibit comparable or better activity to Pt by reducing the amount of Pt metal or using metals that are more abundant on earth. Enhancing catalytic activity by increasing the surface area of Pt is a key strategy for minimizing Pt usage [11]. A higher surface-to-volume ratio leads to an increased surface area of Pt nanoparticles (NPs), which allows for better exposure of catalytic sites [12,13]. Additionally, the tuning of the size, shape, and surface properties of Pt NPs can be achieved with the controlled synthesis of Pt which optimize their catalytic performance for specific reactions [14]. The synthesis and engineering of Pt NPs hold great potential for addressing the challenges related to cost and stability. Utilizing supporting materials to enhance the surface/volume ratio is a well-established strategy in the literature [15–17]. Various supporting materials such as metal oxides [18], polymers [19], carbon black [20], and metal organic frameworks [21] are used to increase surface to volume ratio. Tungsten oxide (WO_3_) has gained interest as a promising support material for metal nanoparticles due to its distinctive properties [18]. The first of its important features is its high surface area and thermal stability, which provides a solid platform for stabilizing Pt NPs [22]. The second noteworthy feature of WO_3_ is that it exhibits redox properties that facilitate charge transfer processes and facilitate the adsorption of reactant molecules [23]. Moreover, combination of Pt NPs with WO_3_ can result in synergistic effects and this enhances the catalytic activity by promoting the spillover of reactants and intermediates in the medium [24,25].

The usage of WO_3_ as a supporting material for Pt NPs provides an improvement of activity and stability while overcoming the limitations of Pt particularly for HER applications.

In this work, the catalyst was obtained via successful impregnation of Pt NPs on WO_3_ using a simple impregnation-reduction method at room temperature. The obtained platinum-loaded tungsten oxide (Pt/WO_3_) catalyst was analyzed by advanced techniques such as XRD, FESEM, TEM, XPS, and ICP. These characterizations verified the uniform dispersion of Pt nanoparticles on the WO_3_ surface and confirmed the successful synthesis. The resulting Pt/WO_3_ catalyst exhibits exceptional HER activity, with an onset potential of −27.8 mV vs. RHE and a low overpotential of −37.4 mV at a current density of 10 mA·cm^−2^. Additionally, the Tafel slope of 68.6 mV.dec^−1^ suggests efficient reaction kinetics following the Volmer-Heyrovsky mechanism. The article presents the results of the Pt/WO_3_ catalyst’s stability and activity in HER, comparing them with relevant findings from the literature.

## 2. Materials & methods

### 2.1. Materials

WO_3_ (BET surface area = 10 m^2^g^−1^) as the support material, chloroplatinic acid (H_2_PtCl_6_) as the precursor for platinum (Pt) nanoparticles and sodium borohydride (NaBH_4_) (98%) as reducing agent were purchased from Sigma-Aldrich.

### 2.2. Synthesis of catalyst

18 mg of Pt precursor, chloroplatinic acid (H_2_PtCl_6_.6H_2_O), and 0.5 g of commercial WO_3_ were dissolved in 100 mL distilled water and stirred for 24 h at RT to allow for the deposition of Pt^2+^ cations onto the WO_3_ surface. After that, the Pt-loaded WO_3_ support is subjected to a reduction process using NaBH_4_ as a reducing agent. Following reduction, the Pt/WO_3_ catalyst is rinsed with distilled H_2_O and dried to eliminate any remaining residues. The obtained catalyst is then characterized using ICP-OES, XRD, FESEM, TEM, and EDX to confirm the successful synthesis and distribution of Pt NPs on the WO_3_ supporting material.

### 2.3. Electrode fabrication and electrochemical analysis

Glassy carbon electrodes (GCEs) were modified using a Pt/WO_3_ suspension. Specifically, 60 mg of Pt/WO_3_ was dispersed in 400.0 μL of isopropanol and sonicated for 1 h. A 2.0 μL aliquot of this suspension was then deposited onto pre-cleaned GCEs without the use of any binders, such as Nafion. The modified electrodes were allowed to dry at room temperature for 1 hour before electrochemical tests. All electrochemical experiments were conducted using an Ivium potentiostat-galvanostat. The counter, working, and reference electrodes were a graphite rod, a GCE (d = 3.0 mm), and a saturated Ag/AgCl electrode (in 3 M NaCl solution), respectively. The GCEs were polished using an alumina suspension (0.05 μM) to ensure proper cleaning before modification.

The catalytic performance of the Pt/WO_3_ was assessed in a 0.5 M H_2_SO_4_ solution using linear sweep voltammetry (LSV) at a scan rate of 5 mV/s. All potential values were referenced to the reversible hydrogen electrode (RHE). The conversion from Ag/AgCl to RHE was determined using the equation [26]:


$$E_{vs,RHE}=E_{vs.\;Ag/AgCl}+0.210+(0.059\times pH)$$

Electrochemical impedance spectroscopy (EIS) was performed within a potential range of 270 to 300 mV vs. Ag/AgCl, covering a frequency range of 0.1–100,000 Hz with a 10 mV amplitude. To evaluate the long-term stability of Pt/WO_3_, chronopotentiometry was conducted at a constant current density of 0.7 mA in a 0.5 M H_2_SO_4_ solution.

## 3. Results and discussions

**Figure 1:**
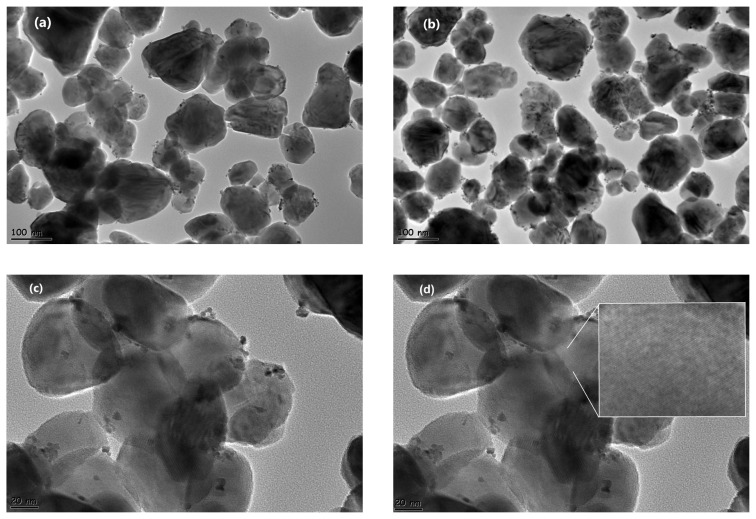

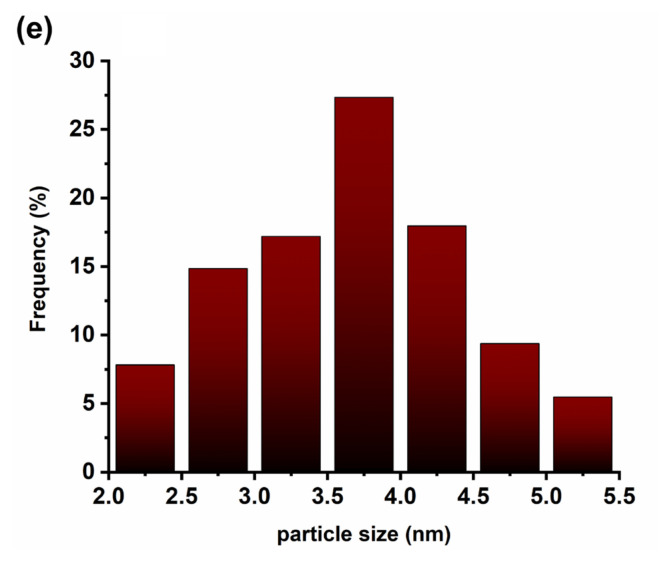
(a), (b) TEM images of Pt/WO_3_ nanoparticles with scale bars of 100 nm and 20 nm. (c) and (d) TEM images of Pt/WO_3_ nanoparticles with scale bars of 100 nm and 20 nm. Inset figure of image (d) shows the lattice fringe of WO_3_. (e) Particle size distribution histogram.

Pt NPs were obtained using a simple impregnation and reduction method onto WO_3_ support at RT. The resulting catalyst was analyzed with ICP-OES to determine the Pt content and confirmed that wt.% Pt/WO_3_ catalyst contained 1.0 wt.% Pt. The theoretical Pt loading (1.3 wt.%) was calculated based on the initial amount of Pt precursor added during the synthesis. Experimental value is slightly lower than the theoretical loading of 1.3 wt.%, representing the loss of the initial Pt precursor. This discrepancy can be attributed to several factors inherent in the impregnation and reduction method. During the washing steps, less strongly anchored Pt nanoparticles may have been lost in the washing solution. Additionally, the relatively low surface area of WO_3_ limits the number of available anchoring sites, potentially leading to the loss of unbound Pt precursors during washing. Furthermore, H_2_PtCl_6_.6H_2_O has a high hygroscopic nature, which can cause partial liquefaction during weighing, potentially affecting the accuracy of the measured precursor mass. Despite the slight deviation, the 1.0 wt. % Pt content is considered acceptable for achieving the desired catalytic activity, and the close agreement between the theoretical and measured values confirms the successful deposition of Pt onto the WO_3_ support. TEM analysis was also performed to investigate the morphology, dispersion and size distribution of Pt/WO_3_ catalyst. Figure 1 presents TEM images of Pt/WO_3_ nanoparticles at different magnifications, providing insights into their morphology and size distribution. Images (a), (b), and (c) display the nanoparticles with scale bars of 100 nm and 20 nm, allowing for a detailed examination of their structural characteristics at varying resolutions. The lower-magnification Figure 1a and b offer an overview of particle dispersion, while the higher-magnification images Figure 1c reveal finer structural details. The inset of the TEM image in Figure 1d reveals clear lattice fringes corresponding to WO_3_, providing direct evidence of its crystalline nature. Additionally, Figure 1e illustrates the particle size distribution histogram, which was generated based on the nanoparticles observed in Figure 1a and b, offering a statistical representation of their size variations. As shown in Figure 1, the obtained TEM images revealed well dispersed and uniform Pt NPs on WO_3_ with approximately 3.7 nm average particle size. The particle size histogram exhibits a narrow range with a low standard deviation (0.7 nm), indicating a homogeneous particle size distribution. This uniformity suggests consistent results across different catalytic tests and repeated measurements. While the reduction in particle size directly increases the total surface area, the uniform dispersion of Pt NPs enhances reactant accessibility by ensuring that more active sites remain exposed and available for catalysis. This improved accessibility contributes to more efficient utilization of the catalyst and enhances catalytic performance.

**Figure 2:**
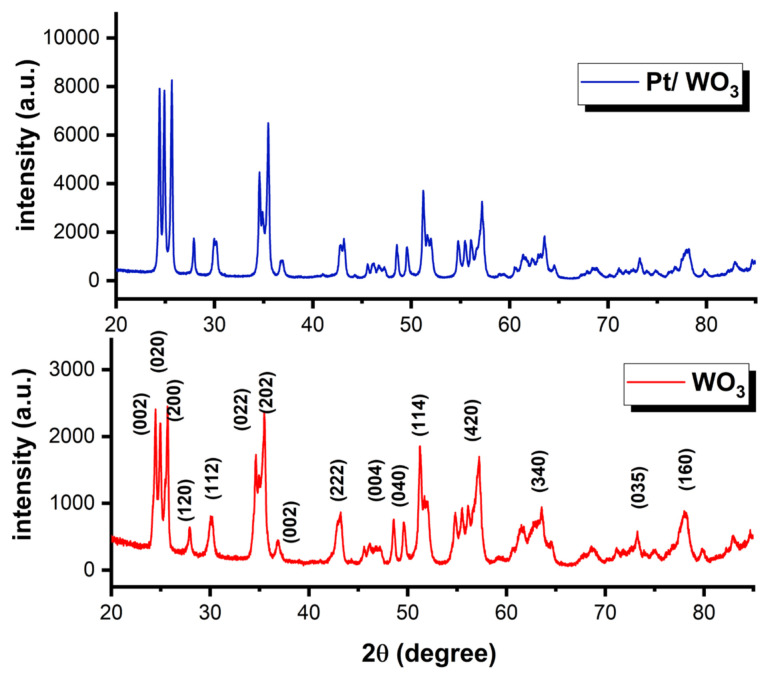
XRD patterns of the Pt/WO_3_ and WO_3_ (JCPDS card No. 83-0950).

For the investigation of the crystalline structure of the synthesized Pt/WO_3_ catalyst, the XRD patterns were investigated. According to XRD analysis that shown in Figure 2, there are characteristic diffraction peaks at 2θ angles of 24.45^o^, 25.19^o^, 25.74^o^, 27.75°, 30.14°, 34.37°, 35.65°, 36.94°, 43.19°, 48.50°, 49.62°, 51.27°, 57.32°, 63.39°, 73.30°, and 78.08° corresponding to the (002), (020), (200), (120), (112), (022), (004), (040), (114), (420), (340), (035), and (160) crystal planes of WO_3_, respectively. These peaks matched well with the peaks belonging to monoclinic WO_3_ (JCPDS card no. 083-0950) [27]. The XRD patterns indicate that there is no observable difference in the crystallinity of WO_3_ after the impregnation and reduction of Pt, confirming that the crystal structure of WO_3_ was preserved throughout the synthesis process. Since the amount of Pt remained below the detection limit of the XRD device due to extremely low Pt loading, additional peaks corresponding to metallic Pt were not detected [18]. However, the presence of Pt was proven using ICP-OES, TEM-EDX, and FE-SEM-EDX spectra.

**Figure 3:**
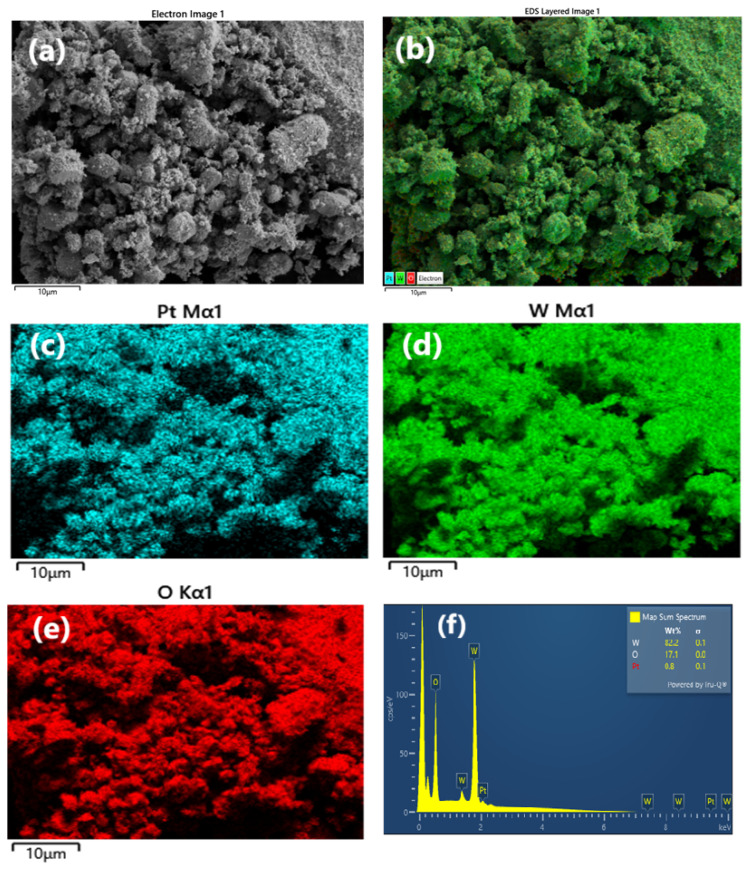
(a) FE-SEM image of the Pt/WO_3_ catalyst. (b–e) the elemental mapping obtained from EDX analysis: (b) the overlay of all detected elements (Pt, W, and O); (c) the distribution of platinum; (d) distribution of tungsten; and (e) the distribution of oxygen. (f) the EDX spectrum, confirming the presence of Pt, W, and O with distinct peaks for each element in the Pt/WO_3_ catalyst.

The FE-SEM analysis was also conducted to investigate the presence and uniformity of Pt NPs in the Pt/WO_3_. The FE-SEM image in Figure 3a shows that the surface exhibits a rough surface morphology with well-distributed particles. Elemental mapping images (Figures 3b–3e) demonstrate that Pt, W, and O are evenly distributed throughout the material. The EDX spectrum (Figure 3f) further confirms the presence of Pt, W, and O, reinforcing the successful synthesis of the Pt/WO_3_ catalyst. Note that the Pt amount detected in the FE-SEM EDX analysis is lower than that measured by ICP-OES due to the limited surface area analyzed by EDX. While ICP-OES provides a bulk measurement of Pt content on the WO_3_ surface, the EDX analysis is more sensitive to a smaller, localized area and, therefore, yields a slightly lower value of 0.8 wt.% Pt loading compared to the 1.0 wt.% obtained from ICP-OES.

**Figure 4:**
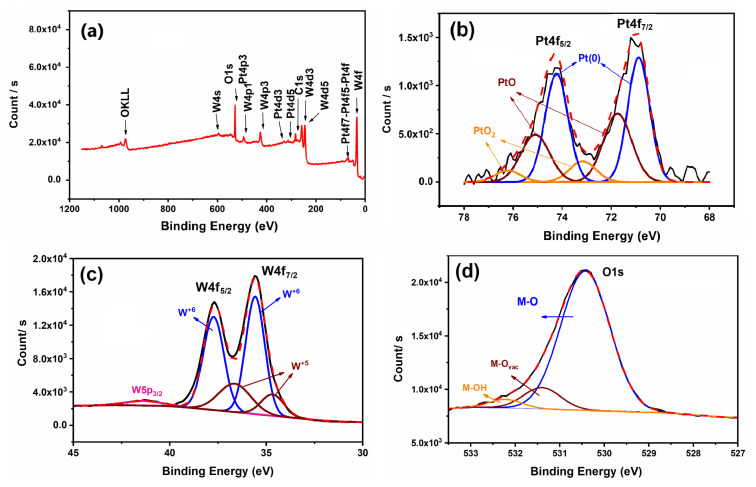
XPS survey spectrum of Pt/WO_3_ catalyst (a) and partial XPS analyses of Pt4f (b), W4f (c), and O1s (d).

The XPS survey spectrum (Figure 4a) provides an elemental composition analysis of the Pt/WO_3_ catalyst. The spectra were calibrated using the C1s peak at 284.8 eV as an internal reference to ensure accurate binding energy measurements [28]. The spectrum confirms the presence of Pt, W, and O, with distinct peaks corresponding to the binding energies of these elements. The high-resolution XPS spectrum for Pt4f (Figure 4b) reveals two primary peaks at 70.88 eV and 74.23 eV, which correspond to Pt 4f_7/2_ and Pt 4f_5/2_, respectively. These peaks indicate the presence of metallic Pt (Pt^0^) and oxidized platinum species (Pt^2+^, Pt^4+^). The observed spin-orbit splitting (ΔE = 3.35 eV) between these peaks further confirms the presence of metallic Pt, in agreement with standard literature values [29,30]. The deconvolution suggests a mixture of oxidation states, which plays a crucial role in the catalyst’s electronic properties. The peaks at 71.74 eV and 75.09 eV suggest the presence of partially oxidized Pt species (Pt^2+^ or Pt-O interactions), while the peaks at 76.21 eV and 79.30 eV indicate the existence of higher oxidation states, such as Pt^4+^ (PtO_2_-like species). The presence of partially oxidized Pt species can be attributed to exposure to air during sample preparation, storage, or XPS analysis can contribute to the oxidation of platinum, as oxygen adsorption on the surface may lead to the formation of PtO (Pt^2+^) and PtO_2_ (Pt^4+^). The XPS analysis of the W 4f region (Figure 4c) indicates the presence of tungsten in multiple oxidation states. The dominant peaks at 35.5 eV (W 4f_7/2_) and 37.7 eV (W 4f_5/2_) correspond to W^6+^ species (WO_3_), confirming the presence of fully oxidized tungsten [31]. The peak at 34.6 eV and 36.7 eV suggests the existence of W^5+^ species (defect-rich WO_3_), which may result from partial reduction due to oxygen vacancies or interaction with the platinum catalyst [31]. The coexistence of W^6+^ and W^5+^ states suggests a complex electronic structure, which can significantly influence catalytic performance by modifying charge transport and oxygen mobility within the system. The O1s XPS spectra (Figure 4c) were deconvoluted into three distinct peaks corresponding to different oxygen species, identified by binding energies of 530.4, 531.4, and 532.2 eV. The peak at 530.4 eV is attributed to oxygen in metal-oxide (M-O) environment, while the 531.4 eV peak corresponds to oxygen vacancies (Vo) within the structure [32]. The higher binding energy peak at 532.2 eV is associated with loosely bound oxygen species, such as hydroxyl groups (M-OH), adsorbed water molecules [32]. These XPS results provide valuable insights into the distribution of Pt, W, and O species on the Pt/WO_3_ catalyst surface.

**Figure 5:**
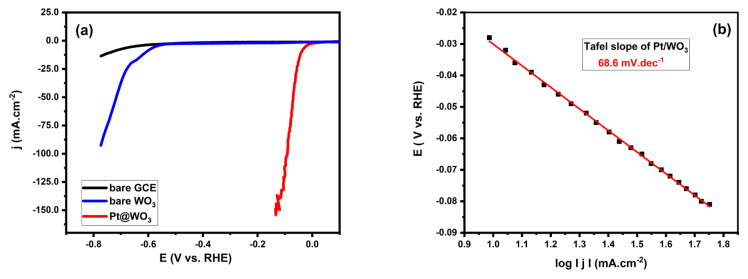
a) Comparative LSV curves of bare GCE, bare WO_3,_ and Pt/WO_3,_ and b) Tafel plot of Pt/WO_3_ catalyst.

After the characterization of the catalyst, electrochemical studies were performed. The LSV analysis was performed to work on the electrochemical characteristics and kinetics of the catalyst towards HER. First of all, the activity of bare GCE and commercial WO_3_ was studied for comparison purposes, and the resulting LSV curves are given in Figure 5. According to the LSV results, bare GCE showed nearly a baseline response, which indicates the lack of catalytic activity. On the other hand, LSV curve for WO_3_ modified GCE exhibits catalytic activity, however, onset potential (h_0_) which is the potential at which the current starts to deviate significantly from the baseline and overpotential to obtain 10 mA.cm^−2^ current density (h_10_) for HER were found as −566 and −610 mV vs. RHE, respectively. The bare WO_3_ shows significantly lower catalytic activity when compared to the Pt/WO_3_ catalyst. Pt/WO_3_-modified GCE exhibits near zero onset potentials at −27.8 mV vs. RHE and −37.4 mV vs. RHE over potential to achieve a current density of 10 mAcm^−2^, which is often used as a benchmark to determine the catalytic performance. These results indicate the promotion of HER activity due to the synergetic effect between the Pt and WO_3_.

Moreover, the Tafel slope (*b*) is another important parameter to understand the electrochemical reaction kinetics and mechanism of the reaction and can be derived from the linear region of the applied potential vs log | j | graph. A lower *b* values indicate faster kinetic and more efficient HER activity. For the Pt/WO_3_ modified GCE, b was found to be 68.6 mV.dec^−1^ (Figure 5b). This value reflects the favorable reaction kinetic and efficient evolution of hydrogen. For the bare WO_3,_ *b* value was found as 120.5 mV.dec^−1^ and which also indicates the positive effect of metal support interaction.

Pt/WO_3_ modified GCE produces −10 mA cm^−2^ at η = 37.4 mV and has 68.6 mV.dec^−1^ Tafel slope with only 42.9 μg.cm^−2^ loading density of Pt. According to these results, Pt/WO_3_ shows high activity for HER as compared to most of the other reported Pt based catalysts given in Table 1. Although some catalysts, such as entries 1, 3, 4, 5, 6, 8, 9, and 10 in Table 1 demonstrate even higher electrocatalytic activity, Pt/WO_3_ offers significant advantages in terms of synthesis and environmental impact. Unlike catalysts that require high-temperature annealing (800–900 ºC) or involve harsh preparation methods using environmentally harmful chemicals, Pt/WO_3_ can be synthesized through a straightforward impregnation and reduction process, ensuring reproducibility and ease of preparation. Additionally, electrocatalytic measurements in this study were conducted without the use of Nafion, a commonly employed polymer for catalyst attachment on GCE, which can introduce additional resistance, interfere with charge transfer and cover the electroactive surface area. Instead, Pt/WO_3_ was directly applied to the GCE via a simple drop-casting technique. Further comparisons can be made between Pt/WO_3_ and other Pt-containing catalysts in terms of their HER activity. For instance, Wang et al. demonstrated that single platinum atoms immobilized on monolayer WO_3_ nanosheets exhibit a low Tafel slope of approximately 27 mV.dec^−1^ [33]. This finding is consistent with the well-established understanding that Pt is among the most effective catalysts for the HER. However, the synthesis of Pt single-atom catalysts on monolayer tungsten trioxide (Pt-SA/ML-WO_3_) necessitates a calcination step at 500 ^°^C to achieve optimal performance. In another study, Jiang et al. reported a heterostructure of Ru_2_P/WO_3_ that significantly improved water dissociation and hydrogen desorption, achieving an overpotential of only 15 mV at a current density of 10 mAcm^−2^ however, annealing at 750 °C is required to complete synthesis [34]. Chen et al. developed Pt/N-CoWO_3_ catalyst via hydrothermal method and thermal annealing at 500 ºC, achieving high stability for acidic hydrogen production requiring the overpotentials of 94 mV to achieve the current densities of 1 A cm^−2^ over extended periods [35]. Pt/WO_3_-400 demonstrates a slightly higher overpotential (57 mV vs. RHE) and Tafel slope (66 mV.dec^−1^) to Pt/WO_3_, suggesting similar HER activity [36]. However, the preparation method of Pt/WO_3_ which is synthesized in this study, offers notable advantages in terms of ease and environmental friendliness. The straightforward synthesis procedure ensures simplicity and reproducibility, facilitating the scalability of Pt/WO_3_ production. Additionally, the environmentally friendly nature of the synthesis method aligns with the growing demand for sustainable catalyst synthesis. Furthermore, the low Pt content in Pt/WO_3_ is a significant advantage (Table 1). The utilization of a minimal amount of Pt not only contributes to cost reduction but also addresses the limited availability and high cost of Pt.

HER mechanism involves two main steps. The first one is the adsorption of the reactants, and the second one is the desorption of the products. The initial electrochemical reduction of water molecules is named Volmer step, and the subsequent electrochemical desorption of hydrogen atoms is named as Heyrovsky (or Tafel) step [16,57]. In the Volmer step, water molecules are reduced to form adsorbed hydrogen atoms on the electrode surface and in the Heyrovsky step, the adsorbed hydrogen atoms combine to form molecular hydrogen gas.


$$\begin{array}{l} {\text{H}}_{3}{\text{O}}^{+}+{\text{e}}^{-}+\text{M}⇌{\text{MH}}_{\text{ads}}+{\text{H}}_{2}\text{O}\;(\text{Volmer}\;\text{step}\;\text{in}\;\text{acidic}\;\text{condition})\\ {\text{MH}}_{\text{ads}}+{\text{H}}_{3}{\text{O}}^{+}+{\text{e}}^{-}⇌\text{M}+{\text{H}}_{2}+{\text{H}}_{2}\text{O}\;(\text{Heyrovsky}\;\text{step}\;\text{in}\;\text{acidic}\;\text{condition}) \end{array}$$

The experimentally determined Tafel slope of approximately 69.7 mV.dec^−1^ shows that neither the Volmer nor the Heyrovsky step is solely rate-determining. The rate determining step of the HER when using Pt/WO_3_ catalyst can be mostly the desorption of the product which is the Heyrovsky step, and the electrochemical reduction of H_2_O molecules to the surface (Volmer step) also affects the kinetics of the reaction because in literature ~120 mV.dec^−1^ Tafel slope is attributed to the Volmer step and ~40 mV.dec^−1^ Tafel slope is attributed to the Heyrovsky step [16,58].

In literature, recent work by Napporns’s group [59] on the chemical exfoliation of bulk MoS_2_ via lithium intercalation provides a compelling parallel to our results. In their study, bulk MoS_2_ in 0.5 M H_2_SO_4_ showed a Tafel slope of 151 mV.dec^−1^, while exfoliated MoS_2_ samples prepared using different lithium reagents (Me-Li,n-Bu-Li, andt-Bu-Li) exhibited significantly lower slopes of 102, 96, and 94 mV.dec^−1^, respectively. These values fall between the ideal Tafel slopes for the Volmer and Heyrovsky steps, indicating that the HER kinetics in these exfoliated materials are governed by a mixed Volmer–Heyrovsky mechanism. Importantly, the efficiency of the exfoliation process and consequently the HER performance was found to follow the order: Me-Li<n-Bu-Li<t-Bu-Li. This trend is attributed to the stability of the generated anions (R^−^), where more stable anions (with the order n-Bu-Li>t-Bu-Li>Me-Li) provide a higher concentration of available Li^+^, leading to an efficient intercalation. The improved intercalation enhances the catalyst’s structural and electronic properties, thereby optimizing the balance between the adsorption (Volmer) and desorption (Heyrovsky) steps. In another work, Menezes et al. (2021) reported that the Pt (111) surface requires a lower activation enthalpy in acidic media compared to alkaline conditions [60]. They observed that in acidic media, a typical Tafel slope of around 74 mV.dec^−1^ indicates that the HER follows a Volmer–Heyrovsky mechanism, where both the adsorption (Volmer) and desorption (Heyrovsky) steps contribute to the overall kinetics. In contrast, they highlighted that alkaline media often exhibit significantly higher Tafel slopes (~150 mV.dec^−1^) due to the additional energy barrier imposed by water dissociation during the Volmer step. Štrbac et al. (2012) investigated the HER mechanism on Pd/Au (111), pure Au (111), and pure Pd electrodes [61]. They reported that HER on pure Au(111) follows the Volmer/Heyrovsky mechanism, characterized by a Tafel slope of 110 mV dec^−1^, indicating balanced contributions from both adsorption and desorption steps. In contrast, pure Pd, due to hydrogen absorption and an undefined surface structure, showed a Tafel slope of 40 mV.dec^−1^, suggesting a Volmer/Heyrovsky mechanism with the Volmer step as the rate-determining step. Interestingly, Pd/Au(111) surfaces exhibited a lower Tafel slope of 70 mV.dec^−1^, which the authors attributed to lateral interactions between adsorbed hydrogen atoms. This result indicated a Volmer/Heyrovsky mechanism with a slow Volmerstep under modified adsorption conditions. These findings highlight the impact of surface modifications on the HER pathway and kinetics.

**Figure 6:**
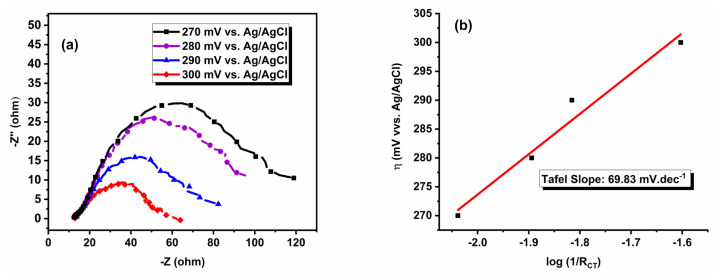
(a) Nyquist plots showing the impedance response at various overpotentials (inset: equivalent circuit model used to fit the experimental data), (b) Tafel plot derived from EIS data.

EIS is an important method to investigate the electrocatalytic behavior of the catalyst and its charge transfer characteristics during the HER. The EIS analyses were performed in the range of 270 and 300 mV vs. Ag/AgCl for Pt/WO_3_ catalyst and the resulted Nyquist plots were given in Figure 6a. Two semicircles can be seen in the Nyquist plots, and each represents the different electrochemical process. To better interpret these processes, an equivalent circuit model (inset of Figure 6a) was used to fit the experimental data. In this model, constant phase elements (CPE) were incorporated instead of conventional capacitors to account for variations in surface roughness and the nonuniform distribution of active sites. The solution resistance (R_s_), representing the resistance of the electrolyte and other ionic pathways, was consistently measured at approximately 13 Ω in all experiments. The first and smaller semicircle, observed in the high-frequency region, corresponds to the contact resistance (R_1_) between the electrode surface and the catalyst, which arises from surface porosity, adsorbed species, or catalyst-electrode interactions [26]. This resistance remains relatively stable across different overpotentials, with values ranging between 15.0–17.0 Ω, indicating a consistent interface between the catalyst and electrode. The other semicircle, appearing at the low-frequency region, expresses the charge transfer resistance (R_CT_) at the catalyst-electrolyte interface, which directly influences HER kinetics. As shown in Figure 6 and Table 2, R_CT_ exhibits a significant decrease with increasing overpotential, dropping from 109.3 Ω at 270 mV to 40.1 Ω at 300 mV, confirming an enhancement in charge transfer efficiency at higher applied potentials. This trend suggests that as the overpotential increases, the catalytic sites become more active, facilitating more efficient electron transfer and improving HER kinetics.

The relation between the h vs. log (1/R_CT_) is also an important analysis for the determination of b value of the catalyst. This relation depends on the assumption that the R_CT_ is the dominant factor contributing to the h. The reciprocal charge transfer resistance (1/R_CT_) is a measure of the ease with which charge transfer occurs at the electrode-electrolyte interface. A lower charge transfer resistance indicates a more facile charge transfer process and therefore a lower overpotential. By plotting overpotential vs log(1/R_CT_), a linear relationship is often observed. This linear relationship is also known as the Tafel plot. As given in Figure 6b, by plotting the overpotential versus log(1/R_CT_), a Tafel slope of 69.7 mV.dec^−1^ was obtained, which closely matched the value derived from the LSV experiments, confirming the consistency of the results. This strong agreement confirms the reliability of the impedance-based evaluation in characterizing the HER kinetics of the Pt/WO_3_ catalyst.

**Figure 7:**
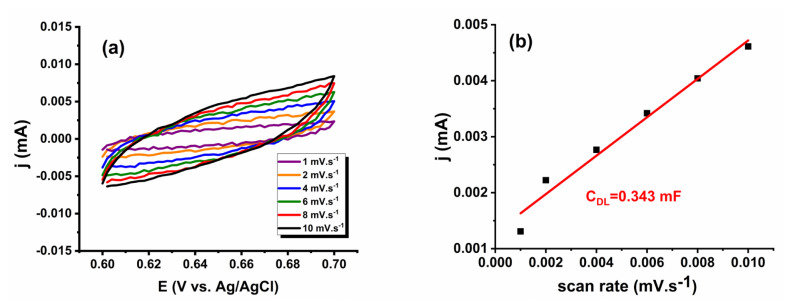
(a) Multiscan CV curves recorded at different scan rates for Pt/WO_3_ and (b) the plot of current versus scan rate used to extract C_DL_.

To determine the ECSA of the catalyst, multicycle CV experiments were carried out at scan rates ranging from 2 to10 mV.s^−1^ in the nonfaradaic region (0.6–0.7 V vs. Ag/AgCl) and given in Figure 7a. The anodic current densities were plotted against the corresponding scan rates in Figure 7b, resulting in a linear relationship with a slope corresponding to the double-layer capacitance (C_DL_) of 0.343 mF. Assuming a specific capacitance (Cs) of 40 μF.cm^−2^ for a flat electrode [62], the ECSA of the Pt/WO_3_ catalyst was calculated to be 8.575 cm^2^. These findings underscore the highly active surface area of the Pt/WO_3_ catalyst, contributing to its enhanced HER performance.

**Figure 8:**
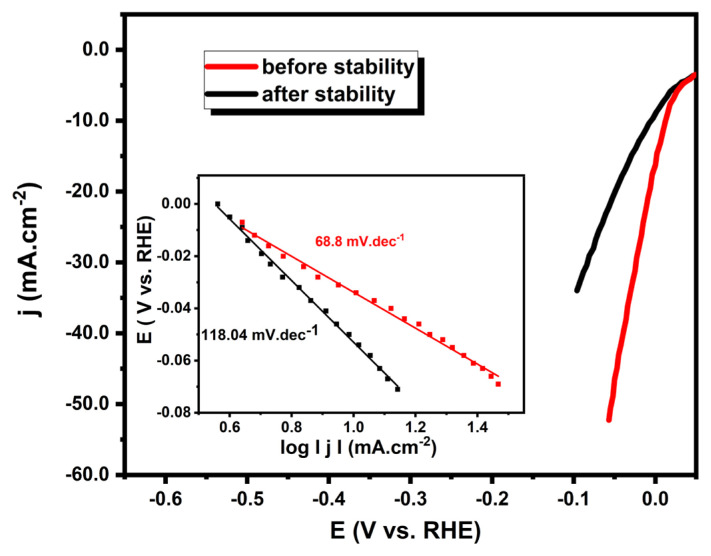
The polarization curves of Pt/WO_3_ at the beginning and after the stability test in 0.5 M H_2_SO_4_. (inset) The corresponding Tafel plots.

The stability of the Pt/WO_3_ catalyst during the HER was evaluated using chronopotentiometry at a constant current of 0.7 mA for 360 s. To assess any changes in catalytic performance, LSV curves were recorded twice: once before the stability test and again immediately after the 360-s chronopotentiometry test, using the same electrode without any regeneration or reuse. As shown in Figure 8, the LSV curve obtained after the stability test indicates a decline in catalytic activity compared to the initial measurement. The inset Tafel plots further quantify this degradation. The *b* value was found as 68.8 mV.dec^−1^ before the stability test and was found to be 118.04 mV.dec^−1^ after the stability experiment. These findings suggest an increase in charge transfer resistance and decrease the activity of catalyst towards HER. The catalytic durability of HER electrocatalysts can be significantly impacted by various factors, such as the leaching of electroactive components due to their insufficient chemical or electrochemical stability, the aggregation of catalyst particles, inadequate mechanical robustness, and so on [63,64]. The performance loss could be attributed to multiple degradation mechanisms occurring at the catalyst surface under HER conditions. The instability can be linked to the formation, growth, and detachment of hydrogen bubbles from the catalyst surface. The accumulation of gas bubbles leads to surface blockage and the formation of dead zones, which increase local electrical resistance and hinder charge transfer [65–67]. In addition to bubble-induced performance loss, catalyst degradation may also cause from structural and compositional changes at the Pt/WO_3_ interface due to the Ostwald ripening effect [64,68,69]. Moreover, prolonged exposure to constant current leads to a mechanical detachment effect caused by bubble evolution. As the bubbles grow and detach from the surface, they generate localized turbulence, which can physically spill catalyst particles from the electrode into the electrolyte [70]. This loss of active material further reduces the electrochemically accessible surface area, contributing to the observed decline in catalytic efficiency. Increasing *b* value reflects possible changes to the surface of the catalyst such as agglomeration or compositional alteration due to the catalyst crumbling off the surface. As a result, these effects collectively contribute to the observed increase in Tafel slope and decrease in catalytic efficiency. Despite the observed stability challenges, the Pt/WO_3_ catalyst demonstrates high HER efficiency with a low onset potential, small overpotential, and favorable Tafel slope. The combination of high catalytic performance, significantly reduced Pt content, and an environmentally friendly synthesis approach highlights its potential as a cost-effective and scalable electrocatalyst for sustainable hydrogen production.

## 4. Conclusion

In conclusion, the Pt/WO_3_ NPs were synthesized successfully with a simple and environment friendly impregnation and reduction method at RT. The synthesized catalyst was characterized by advanced techniques including ICP-OES, TEM, TEM-EDX, XRD, FE-SEM, FE-SEM-EDX and the metal content, dispersion, particle size, and crystallinity were investigated. The Pt/WO_3_ catalyst exhibits well dispersed, uniform Pt NPs with the particle size of ~3.7 nm and which has 1.0 wt.% Pt metal content. Electrochemical studies were also performed, and the results revealed an impressive η_0_ of ~27.8 mV vs. RHE and η_10_ of −37.4mV vs. RHE, showcasing its enhanced catalytic activity. The Tafel slope was also found 68.6 mV.dec^−1^ and suggested efficient HER kinetics, consistent with the Volmer-Heyrovsky mechanism. Furthermore, the catalyst showed robust electrochemical performance and a notable electrochemically active surface area of 8.575 cm^2^, reinforcing its high activity. While stability testing indicated slight degradation over time, the Pt/WO_3_ catalyst retained significant activity. The low Pt content, coupled with a straightforward synthesis approach, positions Pt/WO_3_ as a cost-effective and scalable candidate for sustainable hydrogen production, providing a valuable addition to the development of efficient and eco-friendly electrocatalysts.

## Figures and Tables

**Table 1:** Comparison of some reported electrocatalysts utilized for the HER in 0.5 M H_2_SO_4_.

Entry	Catalyst	Loading density (μg.cm^−2^)	η (mV)	Tafel slope (mV.dec^−1^)	Ref.
**1**	Pt@Cobalt Selenide	-	5@–10	36	[37]
**2**	Rh_2_P/C	3.79	5.4@–5	--	[38]
**3**	PVP-Coordinated Pt	-	10@–10	30	[39]
**4**	Pt Single-Atom Catalyst	-	14@–10	30	[40]
**5**	PtCu/CoP	3.136	20@–10	28	[41]
**6**	CS-PdPt	255	26@–10	33	[42]
**7**	10% Pt/C	75	26@–10	27	[43]
**8**	Pt Nanospheres (Hollow)	-	30.7@–10	23	[44]
**9**	Pt–TiO_2_ NS	16.8	35@–10	33	[45]
**10**	Ni@Pd-Pt	485.9	37@–10	47	[46]
**11**	**Pt/WO** **_3_**	**42.9**	**37.4@**–**10**	**68.6**	**This work**
**12**	Pt/TiO_2_ Nanowire Arrays	-	38@–10	47	[47]
**13**	Pt-FeNi@C	85.7	50@–21	26.1	[48]
**14**	1T-MoS_2_-Pt	-	51@–10	36	[49]
**15**	Pt/VG-SPE	41.9	60@–10	27	[50]
**16**	Rh-MoS_2_	-	67@–10	65	[51]
**17**	Pt@MoS_2_	-	88.43@–10	55.69	[52]
**18**	Ni-Pt film	-	90@–10	41	[53]
**19**	Pt-N_3_O_1_-S_1_	510	92@–400	21.9	[54]
**20**	Pt-MoS_x_/KB-III	-	107@–10	39	[55]
**21**	Pt-Ni@MoS_2_/CC	-	154@–10	121	[56]

**Table 2:** EIS results of Pt/WO_3_ at various overpotentials.

Overpotential (mV)	R_1_ (ohm)	R_2_ (ohm)	R_CT_ (ohm)
270	16.1	125.3	109.3
280	16.9	95.3	78.4
290	17.0	82.3	65.4
300	15.0	55.1	40.1
